# Novel Mito‐Nuclear Combinations Facilitate the Global Invasion of a Major Agricultural Crop Pest

**DOI:** 10.1002/advs.202305353

**Published:** 2024-07-04

**Authors:** Hongran Li, Xinyue Liang, Yan Peng, Zhenxing Liu, Lei Zhang, Ping Wang, Minghui Jin, Kenneth Wilson, Michael R. Garvin, Kongming Wu, Yutao Xiao

**Affiliations:** ^1^ Shenzhen Branch Guangdong Laboratory of Lingnan Modern Agriculture Key Laboratory of Gene Editing Technologies (Hainan) Ministry of Agriculture and Rural Affairs Agricultural Genomics Institute at Shenzhen Chinese Academy of Agricultural Sciences Shenzhen 518000 China; ^2^ State Key Laboratory for Biology of Plant Diseases and Insect Pests Institute of Plant Protection Chinese Academy of Agricultural Sciences Beijing 100193 China; ^3^ School of Life Sciences Henan University Kaifeng 475004 China; ^4^ Lancaster Environment Centre Lancaster University Lancaster LA1 4YQ UK; ^5^ Oak Ridge National Laboratory Biosciences Division, Oak Ridge TN 37830 USA

**Keywords:** energy metabolism, flight performance, genetic composition, mitochondria, *Spodoptera frugiperda*

## Abstract

A fundamental understanding of the underlying mechanisms involved in biological invasions is crucial to developing effective risk assessment and control measures against invasive species. The fall armyworm (FAW), Spodoptera frugiperda, is a highly invasive pest that has rapidly spread from its native Americas into much of the Eastern Hemisphere, with a highly homogeneous nuclear genetic background. However, the exact mechanism behind its rapid introduction and propagation remains unclear. Here, a systematic investigation is conducted into the population dynamics of FAW in China from 2019 to 2021 and found that FAW individuals carrying “rice” mitochondria (FAW‐mR) are more prevalent (>98%) than that with “corn” mitochondria (FAW‐mC) at the initial stage of the invasion and in newly‐occupied non‐overwintering areas. Further fitness experiments show that the two hybrid‐strains of FAW exhibit different adaptions in the new environment in China, and this may have been facilitated by amino acid changes in mitochondrial‐encoded proteins. FAW‐mR used increases energy metabolism, faster wing‐beat frequencies, and lower wing loadings to drive greater flight performance and subsequent rapid colonization of new habitats. In contrast, FAW‐mC individuals adapt with more relaxed mitochondria and shuttle energetics into maternal investment, observed as faster development rate and higher fecundity. The presence of two different mitochondria types within FAW has the potential to significantly expand the range of damage and enhance competitive advantage. Overall, the study describes a novel invasion mechanism displayed by the FAW population that facilitates its expansion and establishment in new environments.

## Graphical abstract

Here, the study describes a novel invasion mechanism displayed by fall armyworm that facilitates its expansion and establishment in new environments. The rice strain used increases energy metabolism, faster wing‐beat frequencies, and lower wing loadings to drive greater flight performance, while corn stran adapts with more relaxed mitochondria and shuttle energetics into maternal investment, observed as faster development rate and higher fecundity.

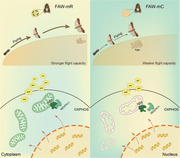

## 1 Introduction

The invasion of non‐native species is a pressing global issue that poses a significant threat to both ecological and food security on a global scale.^[^ ^1^, ^2^ ^]^ Understanding the mechanisms behind biological invasions represents a critical and challenging area of scientific inquiry.^[^ ^3^ ^]^ This field remains a hotbed of research activity with numerous complexities to be explored.^[^ ^4^ ^]^ Efforts to elucidate the factors contributing to the success of certain invasive species have typically concentrated on elements such as population ecology,^[^ ^5^ ^]^ propagule pressure,^[^ ^6^ ^]^ dispersal capabilities,^[^ ^7^ ^]^ adaptability,^[^ ^8^ ^]^ and the evolutionary history of the invaders as well as the invaded ecosystems.^[^ ^9^ ^]^ Some experimental studies have further illustrated the reason from the following perspectives. For example, the loss of intraspecific aggression has been proposed as an explanation for the successful invasion of the Argentine ant (*Linepithema humile*) in the United States.^[^ ^10^ ^]^ The purging of deleterious alleles played a role in enabling the evolution of the globally invasive ladybird beetle (*Harmonia axyridis*), allowing it to maintain high fitness levels even under inbred conditions.^[^ ^11^ ^]^ Moreover, recent investigations have also emphasized instances where invasions were facilitated by hybridization.^[^ ^12^ ^]^

The fall armyworm (FAW), *Spodoptera frugiperda* (J. E. Smith) (Lepidoptera: Noctuidae), is a migratory pest native to the Western hemisphere and has recently invaded the Eastern hemisphere with strong long‐distance flight performance, causing severe damage to multiple agricultural crop species.^[^ ^13^, ^14^, ^15^ ^]^ The FAW first emerged in Africa in 2016 and rapidly spread to 44 African countries in just two years.^[^ ^16^, ^17^, ^18^ ^]^ In May 2018, the FAW was first identified in India, and by December of that year, it had already invaded Bangladesh, Sri Lanka, Myanmar, and other Southeast Asian countries.^[^ ^19^, ^20^ ^]^ The FAW was first reported in Yunnan Province, China in January 2019 and has since spread throughout the country, posing a significant economic threat to China's crop production.^[^ ^21^ ^]^ In February 2020, the FAW was also discovered in Australia and in New Zealand the following year (https://www.fao.org/fall‐armyworm/monitoring‐tools/faw‐map/en/). Due to its global and rapid dispersion and the immense damage it has caused to agricultural production, the invasion mechanism of the FAW has become a topic of considerable interest.^[^ ^22^ ^]^

The FAW from Western Hemisphere can be classified into two subpopulations or strains, namely the “rice” strain and “corn” strain, which are morphologically indistinguishable but differ in host plant distribution and certain physiological features.^[^ ^23^, ^24^, ^25^ ^]^ The “rice” strain primarily feeds on rice and grass species in pasture habitats, while the “corn” strain favors maize and sorghum.^[^ ^26^, ^27^ ^]^ In addition to the distinct host preferences, the two strains also exhibit allochronic differentiation in terms of their mating time and pheromone composition.^[^ ^28^, ^29^ ^]^ A global genetic analysis of the FAW revealed that invasive populations in the Eastern hemisphere were genetically distinct from the American populations, with a highly homogeneous nuclear genetic background.^[^ ^30^ ^]^ Furthermore, through the use of molecular markers such as mitochondrial *COI* (mtCOI) and nuclear *Tpi* genes, as well as genome‐wide analysis, it was discovered that the invasive populations in the Eastern Hemisphere were hybrids between the “rice” and “corn” strains, with a dominant corn‐strain nuclear genetic background.^[^ ^30^, ^31^ ^]^ Consequently, the hybridization in invasive regions possess two mitochondrial haplotypes within a similar nuclear genetic background, referred to as FAW‐mR, which carries the mitochondria from “rice” strain, and FAW‐mC that carries the mitochondria from “corn” strain. The extent to which the phenotypic divergence resulting from the new combination genotypes obtained through inter‐strain hybridization contributes to their invasion success and rapid spread remains poorly understood.

Mitochondria are considered the power plants of eukaryotic cells, producing up to 95% of total adenosine triphosphate (ATP) through oxidative phosphorylation (OXPHOS).^[^ ^32^, ^33^ ^]^ The mitochondrial genome (mitogenome) typically contains 13 protein‐coding genes, which interact with nuclear genes to preform OXPHOS and other functions.^[^ ^34^ ^]^ Since the majority of the proteins required for OXPHOS are encoded by nuclear genes and imported into the mitochondria, transcriptomics provides a powerful tool for studying the complex interactions between nuclear and mitochondrial genes that are necessary for proper mitochondrial function and cellular energy metabolism.^[^ ^35^, ^36^ ^]^ Accumulating evidence has shown that variation in the mitogenome affects mitochondrial function and energy metabolism and thus subsequent biological phenotypes.^[^ ^37^, ^38^ ^]^ For example, three non‐synonymous changes in mitogenome likely increased mitochondrial DNA (mtDNA) copy number, cold tolerance, and fecundity of small brown planthoppers, *Laodelphax striatellus*.^[^ ^39^ ^]^ The standing variation in the mitogenome in Australian *Drosophila melanogaster* can be shaped by thermal selection, and could therefore contribute to evolutionary adaptation under climatic stress.^[^ ^40^ ^]^ The mutations in mtDNA can plausibly affect pre‐copulatory mating success of fruit flies, *D. melanogaster*.^[^ ^41^ ^]^ Given that, we hypothesized that the FAW individuals in Eastern Hemisphere (particularly in Asia) carrying the two types of mitochondria, but with similar nuclear genetic background, may show divergent phenotypes that contributed to their invasion success and rapid dispersal.

Our study involved a multi‐year systematic investigation into the population dynamics of FAW‐mC and FAW‐mR in China from 2019 to 2021. We observed that at the initial invasion stage, the proportion of FAW‐mR individuals was dominant, accounting for over 99% of the population. Following their initial colonization, FAW populations migrated into North China in the spring and then southward again in the autumn.^[^ ^42^ ^]^ In 2021, we conducted a follow‐up survey and monitored newly‐arrived populations in non‐overwintering areas of North China. We found that the proportion of FAW‐mR in the first sample of northward migrating individuals was also higher, accounting for over 98% of the population, while the proportion of FAW‐mR and FAW‐mC in the southern breeding area was nearly equal. Based on these observed field‐based ecological phenomena, we established FAW‐mR and FAW‐mC strains with the similar nuclear genetic background in the lab to test the flight biology and fitness differences and explore the underlying mechanisms underpinning this phenomenon. The results showed that the FAW‐mR used increased energy metabolism, faster wing‐beat frequencies, and lower wing loadings to drive greater flight performance and subsequent rapid colonization of new habitats. In contrast, the FAW‐mC individuals adapted with more relaxed mitochondria and shuttled energetics into maternal investment, observed as faster development rate and higher fecundity. Our findings uncovered a novel invasion mechanism that explains the rapid global expansion of the FAW. This not only advances the theory of “novel mechanisms for the rapid spread of invasive species” but also offers valuable theoretical insights for risk assessment and efficient control of major invasive pests like FAW.

## 2 Results

### 2.1 Dynamics of FAW‐mC and FAW‐mR Strain Across China during 2019–2021

**Figure 1:**
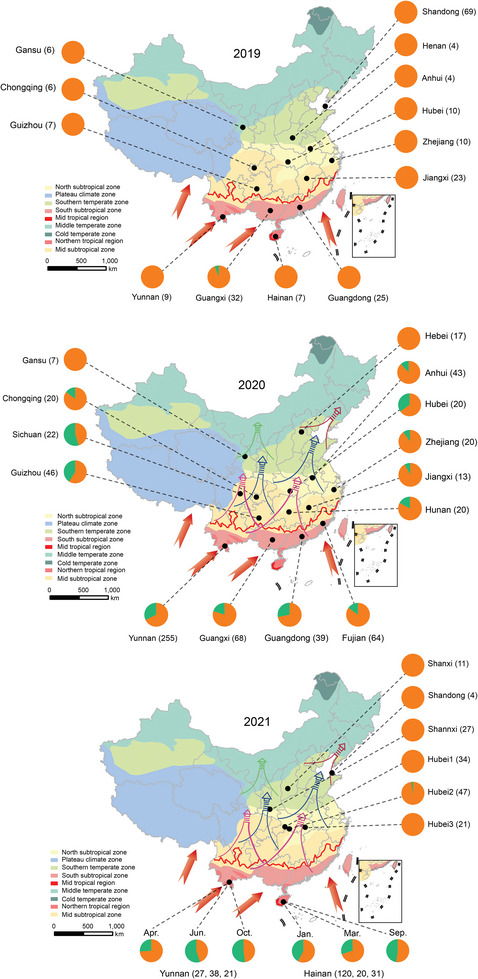
Dynamics of FAW‐mR and FAW‐mC distribution in *Spodoptera frugiperda* across China during 2019–2021. Orange represents the percentage of FAW‐mR strain; green represent the percentage of FAW‐mC strain. The red line in the map represents northern boundary of winter‐breeding area. The arrows out the map represent the possible immigration route from neighboring countries. The arrows in the map indicate the sweeping orientation and areas. A–C) the pie chart north of the overwintering northern boundary shows the proportion of first occurrence of two strains in non‐overwintering area in 2019 and 2021, but not in 2020. The points in the map represent different sampled provinces. The number in bracket represents sample size.

Phylogenetic comparisons using the *mtCOI* gene confirmed that the FAW collected in China during 2019–2021 were mainly identified as the FAW‐mR with a small proportion of FAW‐mC (**Figure** **1**). In the initial invasion of 2019, a total of 214 individuals from 12 provinces were tested and only 2 individuals were identified as FAW‐mC strain, the percent of FAW‐mR was >99% (Figure 1A; Table S1, Supporting Information). In 2020, a total of 654 randomly collected specimens tested, and the percentage of FAW‐mR decreased to 76.2% (156 individuals) (Figure 1B; Table S2, Supporting Information). In 2021, we further conducted a tracing investigation for the first occurrence of FAW in its non‐overwintering area (the areas that FAW must migrate into) in North China. A total of 144 individuals from 6 collections were tested in which FAW was initially discovered in North China. Meanwhile, we also investigated the dynamics of two strains in their overwintering areas (Hainan and Yunnan province) by collecting samples during June to October; the percentage of FAW‐mC strain averaged 42.3%, ranging from 25.9% to 55.2% (Figure 1C; Table S3, Supporting Information). The results showed that the ratio of FAW‐mC and FAW‐mR is almost close to 1:1 in its annual‐breeding area, whereas the first occurrence of FAW that migrated to non‐overwintering areas is almost all FAW‐mR strain (> 98%). In all, we hypothesizes that the FAW‐mR may have a greater cold hardiness so that the northern boundary of the overwintering area is the nearest non‐overwintering area and/or that the FAW‐mR has the stronger long‐distance flight capability, allows them to arrived at non‐overwintering areas earlier than the FAW‐mC.

There were significant differences in the percentage of FAW‐mR among the three years since the first report in China (logistic regression: Χ^2^_1_ = 78.33, P < 0.0001; Figure S1A, Supporting Information). We also investigated the dynamics of FAW‐mC and FAW‐mR since the first report in Africa, which is consistent with our observation in China: the proportion of FAW‐mR decreased with the number of years since FAW was first reported in country at the point of collection in Africa (linear model: F = 13.41, df = 1,36, P = 0.0008) (Figure S1B, Supporting Information). The findings suggest that, in the initial wave of FAW arrivals in China, individuals identified as FAW‐mR may predominate over FAW‐mC, potentially possessing enhanced flight capabilities.

### 2.2 Wing Loading and Flight Performance

The similar nuclear genetic backgrounds of FAW‐mR and FAW‐mC individuals was confirmed in China (Figure S2, Supporting Information). As shown in Figure S2A (Supporting Information), the minimum cross‐validation (CV) error is K = 1. When utilizing the minimum CV error value (K = 1), individuals from China and the USA displayed a similar population structure (indicated by identical colors between them). Further, when using K of 2, a significant population structure was observed among FAW populations from China and America, with those from China sharing a common ancestry (Figure S2B, Supporting Information).

**Figure 2:**
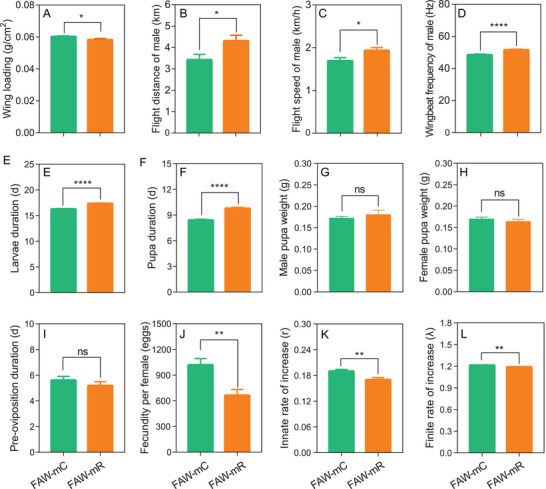
Fitness comparison of FAW‐mC and FAW‐mR strain in *Spodoptera frugiperda*. A) wing loading of FAW‐mC and FAW‐mR strain. B–D) flight capability of FAW‐mC and FAW‐mR strain. E‐L, fitness parameters of FAW‐mC and FAW‐mR strain. “*” “**” “*****” indicate significant differences at P < 0.05, P < 0.01 and P < 0.0001.

To exclude the possible interference of reproduction on flight performance only male adults were used for further studies. Wing‐loading varied between strains, with FAW‐mC having wing‐loadings that were higher than FAW‐mR overall (t = 2.004, df = 164, P = 0.04) (**Figure** **2**A). To investigate the differences in flight performance between the FAW‐mC and FAW‐mR, we recorded the flight parameters during a 3 h flight test. We found FAW‐mR had the longer flight distance (t = 2.39, df = 200, P = 0.02), higher wingbeat frequency (t = 3.86, df = 200, P = 0.0002) and greater speed (t = 2.42, df = 200, P = 0.02) (Figure 2B–D). These results show that in males at least, FAW‐mR moths have the stronger flight performance than FAW‐mC moths.

### 2.3 Cold Hardiness

There was no significant difference in the supercooling point between FAW‐mC and FAW‐mR in 3rd stage larvae (t = 0.92, df = 74, P = 0.36), 5th stage larvae (t = 1.28, df = 55, P = 0.21) or the pupal stage (t = 0.40, df = 97, P = 0.69) (Figure S3, Supporting Information). Similarly, no significant difference was found in freezing point between them in the 3rd larval stage (t = 1.83, df = 80, P = 0.07), 5th larval stage (t = 0.19, df = 55, P = 0.85) or the pupal stage (t = 0.30, df = 93, P = 0.76) (Figure S3, Supporting Information). The results indicate that there was no significant difference in cold hardiness that could explain the relative frequencies of the FAW‐mC and FAW‐mR in the overwintering and non‐overwintering sites.

### 2.4 Fitness Comparison

To clarify whether there was any difference in the relative fitness of the FAW‐mC and FAW‐mR we compared a number of life‐history parameters (Figure 2E–I). The results showed that larval (t = 7.69, df = 198, P < 0.0001) and pupal duration (t = 9.03, df = 180, P < 0.001) of FAW‐mC insects is shorter than that of FAW‐mR, while the weight of FAW‐mC female (t = 0.84, df = 75, P = 0.402) and male pupae (t = 0.69, df = 121, P = 0.489) were not significant with those of FAW‐mR females and males. There was no significant difference in pre‐oviposition period (t = 0.96, df = 74, P = 0.34), but the FAW‐mC females had significantly higher fecundity (1020 eggs per female) than that of FAW‐mR (664 eggs per female) (t = 3.42, df = 73, P = 0.001) (Figure 2E–J). The innate rate of increase (r) and finite rate of increase (λ) of the FAW‐mC strain were significantly higher than that of FAW‐mR (P < 0.01) (Figure 2K,L), at least in this laboratory setting, suggesting that FAW‐mC would outcompete FAW‐mR in the field unless there are any fitness advantages not captured by these life‐history traits.

### 2.5 Variation in Mitochondrial Genome and Morphology

**Figure 3:**
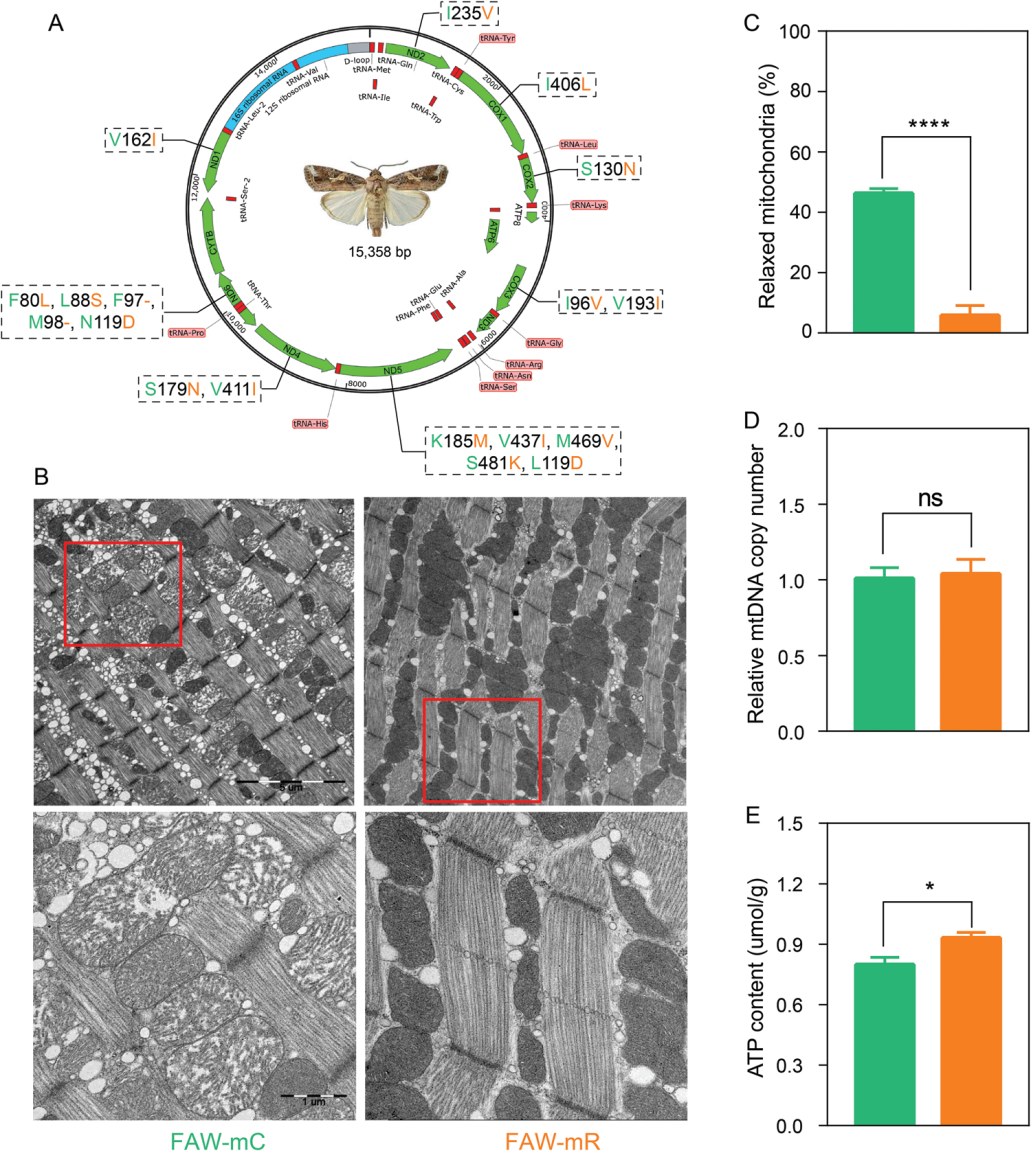
Nonsynonymous variation of mitochondrial genome in *Spodoptera frugiperda*. A) dotted boxes represent number and sites of nonsynonymous variation, blue font represents FAW‐mC strain, whereas red font represents FAW‐mR strain. B) representative TEM images of mitochondrial morphology in flight muscle tissues. The boxed areas were enlarged in the bottom panels. Scale bars, 1 µm (5000×, bottom) and 5 µm (1700×, top). C) percentage of relaxed mitochondria in flight muscle tissues of FAW‐mC and FAW‐mR strain. D) relative number of mitochondria between FAW‐mC and FAW‐mR strains. E, comparison of ATP content between FAW‐mC and FAW‐mR strains (n = 8). Student's *t*‐test were performed for the analysis of (C), (D), and (E) figures. “*” “**” “*****” indicate significant differences at P < 0.05, P < 0.01, P < 0.0001.

We successfully obtained 51 and 53 unique full mitochondrial genomes from FAW‐mC and FAW‐mR individuals, respectively, and further focused on the nonsynonymous mutations in 13 protein‐coding genes (**Figure** **3**A). The total length of the concatenated sequences of 13 mitochondrial protein genes was 11 202 bp, with no indels or premature stop codons being observed. There were 213 variable sites, and the nucleotide diversity was 1.901%. Moreover, 18 non‐synonymous changes located on eight of 13 protein‐coding genes identified a single haplotype that delineates the two strains. Five non‐synonymous changes were identified in the ND5 (amino acid: K185M, V437I, M469V, S481K, L550F) and ND6 (amino acid: F80L, L88S, F97‐, M98‐, N119D) gene, respectively. Two were associated with non‐synonymous variation in the COX3 (amino acid: I96V, V193I) and ND4 (amino acid: S179N, V411I) genes, respectively. A single non‐synonymous change was found in the ND1 (amino acid: V162I), ND2 (amino acid: I235V), ATP8 (amino acid: I1M), COX1 (amino acid: I406L), and COX2 (amino acid: S130N) genes.

To further investigate a link between the strains in mitochondrial activity for the non‐synonymous variation, we observed flight muscle tissues through transmission electron microscopy (TEM). The results showed a clear difference in cristae structure between the two strains in male adults (Figure 3B). In the FAW‐mR strain, the mitochondrial cristae density is highly compacted while it is mainly relaxed in the FAW‐mC strain. The number of relaxed mitochondria in the FAW‐mC strain were significantly greater than the FAW‐mR strain by 42% (Figure 3C; Table S4, Supporting Information, percentage of relaxed mitochondria in male FAW‐mC: 46.28 ± 1.52%; male FAW‐mR: 5.79 ± 3.63%, P < 0.0001). These experimental observations lead us to conclude that difference in mitochondrial structure might change replication and functional efficiency between FAW‐mC and FAW‐mR.

### 2.6 mtDNA Copy Number and ATP Content

To define the mitochondria functionality and metabolic mechanisms that are involved in the flight capability difference, we measured the mtDNA copy number and ATP concentration in the flight muscle of these FAW. We found that there was no significant difference in mtDNA copy number between FAW‐mC and FAW‐mR (t = 0.252, df = 6, P = 0.890) (Figure 3D). However, ATP concentration was significantly greater in the male FAW‐mR strain compared with the FAW‐mC (t = 2.93, df = 14, P = 0.01) (Figure 3E).

### 2.7 Protein Homology Modeling

**Figure 4:**
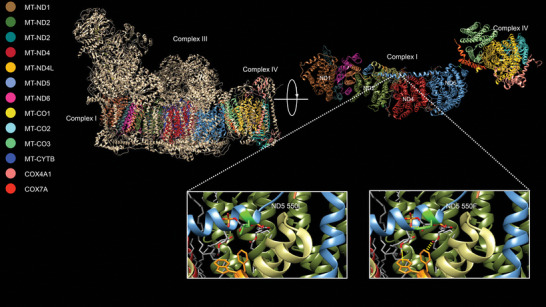
Mitochondrial Supercomplex from Bos taurus (PDB 5xth). All mitochondrial‐ and two nuclear‐encoded subunits are colored according to the key on the left. Nuclear‐encoded subunits were removed and structure was rotated (top right) to reveal the ND5 “piston armature” that contains one of the amino acid changes of interest (ND5 L550F). The ND5 L550F is interesting in that it is predicted to involve interactions between proteins and the phospholipid cardiolipin. Pi‐stacking is a strong molecular force among amino acids with aromatic side chains. These can be between amino acids but also with phospholipids. Here in the structure for complex I it appears two residues in nuclear‐encoded NDUFA11 form pi‐stacking interactions with a cardiolipin molecule that is intertwined with the “piston arm’ of ND5. The substitution of phenylalanine (ND5 550F, FAW‐mR strain) introduces a much larger hydrophobic aromatic side chain which is predicted to introduce pi‐stacking with a cardiolipin molecule.

In order to determine if the amino acid changes in the mitochondrial‐encoded proteins could explain the observed differences between FAW‐mR and FAW‐mC in the laboratory experiments, we performed in silico predictions of their functional effects. From our analyses the substitution of phenylalanine (ND5 550F, FAW‐mR) introduces a much larger hydrophobic aromatic side chain, which is predicted to introduce pi‐stacking with a cardiolipin molecule (**Figure** **4**). This structural prediction is consistent with the higher ATP output and more compact mitochondria observed in the FAW‐mR harboring a 550F. This would be in addition to the pi‐stacking that is likely occurring with the tryptophan and phenylalanine at positions 59 and 60 in the nuclear‐encoded subunit NDUFA11 (Sfru015409.1). The additional stabilization with the third pi‐stacking interaction adds further support for the compact cristae observed in the FAW‐mR strain (550F) compared to the FAW‐mC strain (550L).

### 2.8 Obvious Difference in Energy Metabolism

**Figure 5:**
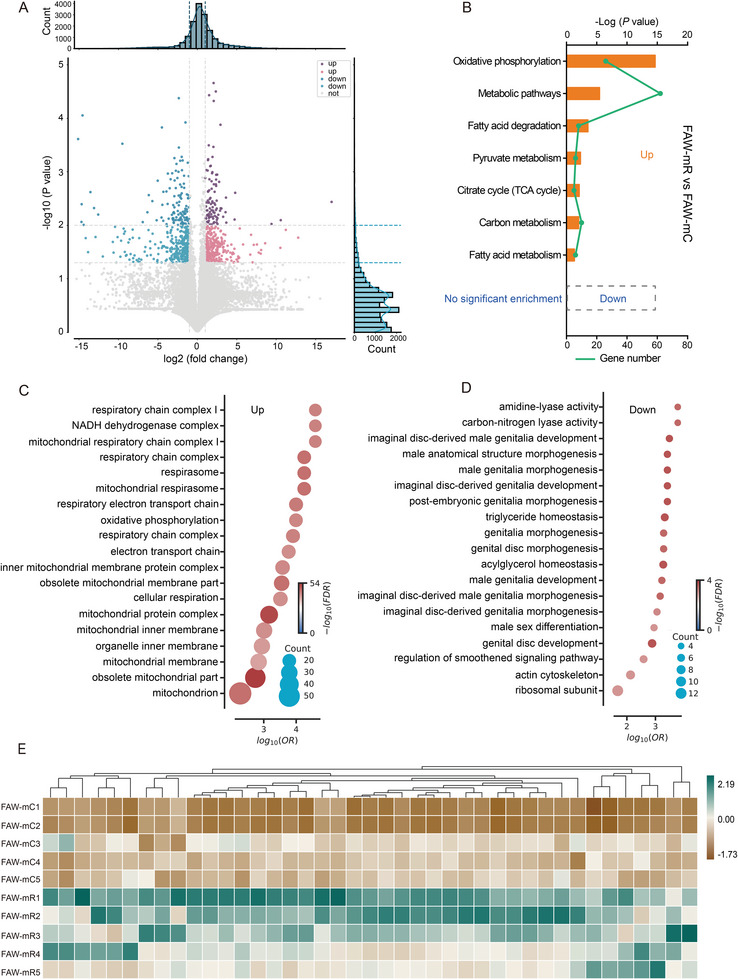
Energy metabolic profile differences between FAW‐mC and FAW‐mR strains in fall armyworm, *Spodoptera frugiperda*. A) Vocano plot of differently expressed genes (DEGs). B) KEGG enrichment of DEGs. Only KEGG terms involving the energy metabolism mainly in mitochondria with adjusted P < 0.05 are shown (*t*‐test). C,D) GO enrichment of DEGs. GO terms involved in mitochondrion activity in FAW‐mR strain, while it is not present in FAW‐mC strain (Figure 5C,D). E) Heat map representing the gene‐expression levels is involved in energy metabolism. The genes represent the different energy metabolism processes, as follows: oxidative phosphorylation, fatty acid metabolism and degradation; pyruvate metabolism, TCA cycle; and carbon metabolism. Heat‐map signal indicates expression level within the group. Green signal represents higher expression, whereas yellow represents lower expression.

To explore the molecular basis of flight trait differentiation we conducted a transcriptomic analysis of flight muscles. A total of 828 DEGs were identified with P < 0.05 between FAW‐mC and FAW‐mR (**Figure** **5**A). Approximately 405 of 828 DEGs were highly expressed in the flight muscles of FAW‐mR, and some of these highly expressed genes were enriched mostly in pathways involved in energy metabolism, including oxidative phosphorylation, carbon metabolism, citrate cycle (TCA cycle), fatty acid degradation, pyruvate metabolism, and fatty acid metabolism according to KEGG signaling pathway analysis (adjusted P < 0.05, *t*‐test). However, the 423 DEGs highly expressed in the FAW‐mC showed no significant enrichment in specific pathways involved in energy metabolism (Figure 5B). Similarly, all DEGs were highly expressed in the flight muscles of FAW‐mR were enriched mostly in GO terms involved in mitochondrion activity, while it is not present in FAW‐mC (Figure 5C,D). Further, we focused on DEGs and pathways related to the energy metabolism that took place in the mitochondria‐nuclear complex. A total of 61 DEGs in mito‐nuclear complex that are involved in oxidative phosphorylation, carbon metabolism, TCA cycle, fatty acid degradation, pyruvate metabolism, and fatty acid metabolism were up‐regulated in FAW‐mR (Figure 5E). Therefore, the most obvious difference between FAW‐mR and FAW‐mC in flight muscles was in terms of energy metabolism.

## 3 Discussion

Interspecific hybridization can lead to the emergence of new evolutionary traits and/or increased genetic and phenotypic diversity, both of which can supply genetic material for rapid adaptation to new environmental conditions.^[^ ^43^, ^44^, ^45^ ^]^ Several instances have been documented where interspecific hybridization has given rise to populations with unique invasive traits. These examples have mostly focused on the genetic benefits of hybridization during invasion, such as heterosis (also known as hybrid vigor)^[^ ^46^ ^]^ or genetic rescue effects,^[^ ^47^, ^48^ ^]^ as seen in the clonal freshwater snail *Melanoides tuberculata* and the invasive lepidopteran pest *Operophtera brumata*. Despite the fact that interspecific hybridization may facilitate the establishment of invasive species in some cases, little is known about the genetic advantages of inter‐strain hybridization within an invasive species during rapid invasion and spread, especially with regards to the effects of mitochondrial import and variation in the mitochondrial genome during hybridization. In China, two populations of the invasive FAW have been found to carry different mitochondrial haplotypes while sharing a homogeneous nuclear genetic background, making them an ideal model to investigate the mechanism of two independent invasion and colonization events.^[^ ^30^ ^]^ Here, we reported that FAW utilized different strategies to adapt to a novel environment in China after their inter‐strain hybridization (e.g., “rice” mitochondria combined with “corn” nuclear genome), and our in silico functional predictions support the hypothesis that the amino acid changes in mitochondrial‐encoded proteins are at least partially responsible for these adaptations, which will consequently expand their range and increase threats to economically important crops.

**Figure 6:**
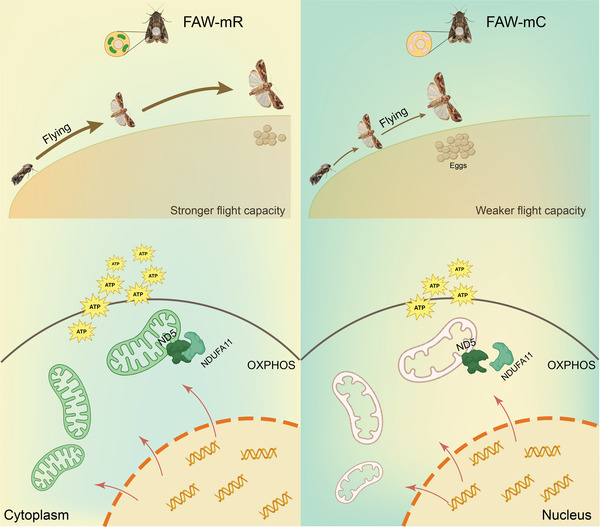
Scheme of a novel invasion strategy diverged in fall armyworm, *Spodoptera frugiperda*.

The inter‐strain hybridization of FAW initially occurred in their native Western Hemisphere, with a small population of hybrid individuals quickly invading the Eastern Hemisphere.^[^ ^31^ ^]^ Despite a highly homogeneous nuclear genomic background, this invasive hybrid group possesses two distinct mitochondrial haplotypes. Our research suggests that given their similar nuclear genetic backgrounds, it is likely the mitochondrial haplotypes that provide their respective advantages, thus enabling successful invasion into new environments and subsequent expansion. This is supported by our laboratory results. As a result, this has led to an improved invasion efficiency and a significant expansion of the northern boundary of FAW infestation in China. Specifically, FAW‐mR display a rapid ability to invade and spread to new habitats through faster flight, while FAW‐mC possess weaker flight capabilities, limiting their ability to spread. However, FAW‐mC possesses superior fecundity and can rapidly increase the population biomass and competitiveness locally. This strategy resembles the military's multi‐arm tactics, where mechanized regiments quickly occupy local control areas, followed by infantry and security forces that secure and clear the occupied region. Our finding suggests a novel combination of nuclear and mitochondrial genomes may facilitate biological invasions (**Figure** **6**).

The novel invasion strategy not only increased the invasion efficiency of FAW, but also expanded its damage range in the new habitat. The fusion of “rice” mitochondria with “corn” nuclear genome notably widened the northern boundary of FAW infestation in China. The primary corn‐growing regions, including the Huang‐Huai‐Hai Summer Corn Region (located mainly in Henan, Shandong, and Hebei provinces) and the Northeast Spring Corn Region (encompassing Liaoning, Jilin, Heilongjiang, and eastern Inner Mongolia), as well as the Korean Peninsula and Japan, are potentially suitable for summer‐breeding populations of FAW.^[^ ^49^, ^50^, ^51^ ^]^ Consequently, the rapid expansion of the FAW population poses a significant threat to Chinese agricultural production and food security.

We discovered that FAW‐mC had a higher fecundity than FAW‐mR, indicating that the adaptive strategy of FAW‐mC would lead to its increased prevalence in the annual breeding area of South China. Nevertheless, our long‐term investigation did not observe this trend. We hypothesize that this could be attributed to the return migration of FAW‐mR individuals from north to south during autumn and winter,^[^ ^42^ ^]^ similar to that seen in FAW in North America.^[^ ^26^ ^]^ Eastern Asia has seen FAW establish a seasonal migration pattern, making it a significant agricultural pest that migrates north to south and back.^[^ ^50^, ^52^ ^]^ This autumnal return migration was enough to counterbalance the population surge caused by the reproductive dominance of FAW‐mC in the year‐round breeding areas, leading to a balance between FAW‐mR and FAW‐mC individuals. The outcome of this balance is not only a range expansion of their habitat but also the assurance of competitiveness of species in the same ecological niche as the year‐round breeding area.

Numerous studies have found that sequence variation in the mtDNA can affect the expression of a range of life‐history and health‐related traits, from fertility to longevity.^[^ ^40^, ^53^, ^54^, ^55^ ^]^ Many of these mtDNA‐mediated effects on phenotype appear to be interactions with the nuclear genome.^[^ ^56^ ^]^ As mtDNAs diverge in different populations of a species, selection should favor coevolved nuclear alleles that help maintain important mitochondrial activities. Generally, four out of five complexes involved in OXPHOS are formed by subunits encoded by both the mitochondrial and nuclear genomes, leading to the expectation of mito‐nuclear coevolution.^[^ ^57^ ^]^ Notably, the FAW‐mC and FAW‐mR are easily identified by different mitochondrial haplotypes that include 18 non‐synonymous variations in the mitochondrial genome. Given that the nuclear genomes are nearly identical, we hypothesized that these mitochondrial changes are responsible for at least some of the phenotypic differences of the two strains. Predictive models of one mutation in ND5 support this.

The mitochondrial‐encoded ND5 protein is one of the four proton pumps in complex I, and has demonstrated a strong signature of evolution in numerous taxa,^[^ ^40^, ^58^ ^]^ most notably the “piston arm” that appears to regulate the movement of protons through the structure.^[^ ^59^ ^]^ The L550F substitution in MT‐ND5 is somewhat conservative and rather than altering protein‐protein interactions, this residue appears to alter interactions with cardiolipin bridged through the nuclear‐encoded NDUFA11 and the unique helix HL of ND5 that traverses the complex and appears to regulate the proton pumps.^[^ ^60^ ^]^ Cardiolipin is an important membrane phospholipid for mitochondrial function as it constitutes ≈20% of the inner mitochondrial membrane, acts as a “proton trap” to maintain the electromotive force, and is instrumental in determining the curvature of the organelles and formation of cristae.^[^ ^61^ ^]^ The substitution of phenylalanine (ND5 550F, FAW‐mR) introduces a much larger hydrophobic aromatic side chain, which is predicted to introduce pi‐stacking with a cardiolipin molecule. This would be in addition to the pi‐stacking that is likely occurring with the tryptophan and phenylalanine at positions 59 and 60 in the nuclear‐encoded subunit NDUFA11 (Sfru015409.1). NDUFA11 is necessary for the assembly of complex I and removal of it abolishes activity.^[^ ^62^ ^]^ The additional stabilization with the third pi‐stacking interaction may explain the more compact cristae observed in the FAW‐mR (550F) compared to the FAW‐mC (550L). Further, the transcriptomic analysis of flight muscles showed that highly expressed genes in FAW‐mR were enriched mostly in pathways involved in energy metabolism while in the FAW‐mC strain did not, which indicated L550F substitution in MT‐ND5 may play a more important role in the energy metabolism.

Indeed, the specific role of the mitochondrial ND5 gene in insect species remains challenging to determine due to limitations in targeted base editing of mtDNA. While recent advancements, only two types of mitochondrial gene bases can be edited. Such as the development of DddA‐derived cytosine base editors (DdCBEs),^[^ ^63^ ^]^ have enabled mtDNA editing in animals^[^ ^64^, ^65^ ^]^ and plants,^[^ ^66^ ^]^ they are primarily limited to C‐to‐T conversions in the 5′‐TC context. Another promising avenue involves TALE‐linked adenine deaminases, which facilitate A‐to‐G editing in human mitochondria.^[^ ^67^ ^]^ These limitations restrict the range of mutations that can be introduced, hindering precise manipulation of mtDNA sequences, including those within the ND5 gene in two strains of the fall armyworm. For example, the substitution mutation of F550L in the ND5 gene involves changing the codon from A to T.

Thus, it's important to note that ATP production in cells primarily occurs through OXPHOS in the mitochondria. OXPHOS involves five protein complexes, four of which are formed by subunits encoded by both the mitochondrial (mtDNA) and nuclear (nuDNA) genomes.^[^ ^68^ ^]^ This shared genetic contribution underscores the concept of mitonuclear coevolution, where nuclear and mitochondrial genomes have evolved in coordination to optimize cellular function.^[^ ^57^ ^]^ Given the limitations in mtDNA editing, future research efforts may transfer to nuclear genes that interact with ND5 to regulate ATP generation. The gene editing of nuclear genes is more mature in insect species, providing a potential avenue for investigating the role of nuclear‐mitochondrial interactions in ATP production and cellular metabolism.

Moreover, seen from the perspective of the FAW‐mC strain, it could be argued that this represents an example of the Mother's Curse.^[^ ^37^ ^]^ Because it is maternally inherited, mitochondrial DNA is subjected to natural selection over time in females but not males. Therefore, deleterious mutations in the mitochondrial genome that affect male traits (e.g., sperm production) will not be purged, especially if those mutations confer an advantage in females (e.g., fecundity and maternal investment). In this study, it is potentially a Mother's Curse scenario in which the higher developmental and reproductive success of FAW‐mC females comes at a cost in terms of male flight. Indeed, the GO enrichment of our differentially expressed genes identified several biological processes associated with male genital development, supporting this hypothesis.

In conclusion, our study used a combination of observational data on the nuclear and mitochondrial genomics of FAW, along with detailed laboratory experiments, to provide compelling evidence of the rapid adaptation of an invasive species in China. The two hybrid strains of FAW employed different invasion strategies to adjust to the novel environment, with FAW‐mR displaying stronger long‐distance migration characteristics to quickly expand the invasion range, while FAW‐mC employed higher fertility to improve its competitiveness in the new habitat, thereby increasing the population size. Furthermore, our analysis of molecular mechanisms revealed that changes in amino acids in mitochondrial‐encoded proteins may have led to differences in energy metabolism levels, which contributed to the division of labor among FAW individuals.

## 4 Experimental Section

### Specimen Sampling and DNA Extraction

Individual FAWs were mainly collected from maize fields as larvae (by hand‐collecting) and moths (by light and pheromone trapping) from March to December during 2019–2021. A total of 1143 individuals (214 in 2019, 654 in 2020, and 401 in 2021) were sampled across China over the three years, covering the majority of the known distribution in overwintering and non‐overwintering areas (Table S1, Supporting Information; Figure 1). In particular, to obtain the first FAWs arriving in its non‐overwintering area in North China, a tracing investigation was performed over the northern boundary of overwintering areas from May to October during 2019 and 2021, the majority of the individual samples that were originally discovered in North China (e.g., Shandong, Shanxi, Hebei, Gansu) were provided through cooperation with several universities, the Chinese Academy of Agricultural Sciences, and local agricultural agencies. However, the FAW samples in 2020 were collected randomly due to the uncontrolled nature of the initial occurrence in northern China that required migration into. The collected larvae were individually maintained in vials with maize leaf while adults were kept dry in a vial. Specimens were then brought to the laboratory and stored either air‐dried or in 95% ethanol in a freezer at −20 °C until DNA extraction.

Given the numerous lepidopteran pests of corn in China that potentially complicates the identification of fall armyworm, the specimen's identity was first confirmed by morphological differences and mtCOI sequence analysis.^[^ ^69^ ^]^ A portion of each specimen was excised and subject to total genomic DNA extraction using the EasyPure Genomic DNA Kit (Transgen Biotech, China) following the manufacturer's protocol.

### Strain Identification of Fall Armyworm

The strains were identified based on the mitochondrial cytochrome oxydase I (mtCOI) gene, using the primer pair COI‐F (5′‐ TTCGAGCTGAATTAGGGACTC −3′) and COI‐R (5′‐ GATGTAAAATATGCTCGTGT −3′) as described by Jia et al. (2021).^[^ ^70^ ^]^ All PCRs were performed using 50 µL samples of a solution containing 19 µL double‐distilled water, 25 µL master mix (Vazyme Biotechnology Co. Ltd., Nanjing, China), 4 µL forward and reverse primer mixture (5 µM each), and 2 µL DNA template. PCR was performed under the following conditions: 94 °C for 5 min; 34 cycles of 94 °C for 30 s, 55 °C for 30 s, and 72 °C for 30 s; and 72 °C for 5 min. The amplified products were separated by 1.5% agarose gel electrophoresis and then directly sequenced bi‐directionally using ABI 3730 DNA analyzer at Sangon Biotech (Shanghai, China). Geneious software version 7.1.4 was used to distinguish the heterozygous sites and obtain the accurate gene fragment sequence.^[^ ^71^ ^]^ The published *mtCOI* sequences of both strains of FAW were downloaded from the GenBank database for comparison, and sequence alignment and cluster analysis were performed using MEGA version 7.0.^[^ ^72^ ^]^

### Isolation and Establishment of FAW‐mR and FAW‐mC

Fall armyworms collected from Nanning, Guangxi province and Sanya, Hainan province, China in 2021 were used to screen the FAW‐mR and FAW‐mC strain. Field‐collected larvae in the mixed populations were reared on an artificial diet (the unpublished feed formula contains wheat germ meal as the main component) until pupation in climate chambers under controlled conditions (25 ± 1 °C, 16 h light/8 h dark, and relative humidity 60 ± 5%). After pupal emergence, adults were provided access to a 10% sucrose solution for nutrition. To establish the FAW‐mR and FAW‐mC strains, ≈60 pairs of FAW were established and examined individually using the *mtCOI* gene fragment described as above. The offspring were subsequently pooled together to create FAW‐mR and FAW‐mC depending on their parents’ COI status. Then, the purity of each isolation was monitored every month by randomly sampling 20 individuals.

To confirm the similarity of nuclear genetic background between the FAW‐mR and FAW‐mC strains, 32 individuals were randomly selected (4 for FAW‐mR in USA, 14 for FAW‐mR and 14 for FAW‐mC in China) in the original population for genome resequencing. The Illumina raw reads from resequenced samples were filtered using clean adapter and clean lowqual software, resulting in high‐quality reads with an average error rate of <0.01. The high‐quality reads were then aligned to the fall armyworm reference genome (American corn strain) and mitochondrial genome sequences using bwa mem software^[^ ^73^ ^]^ version 0.7.5a with default parameters. Alignments for each sample were processed by removing duplicate reads using the SAMTOOLS^[^ ^74^ ^]^ software package version 1.3. The mpileup function in samtools was used to generate mpileup files for each sample. Variant calling was performed for all samples using the Genome Analysis Toolkit (GATK, version 3.6‐0‐g89b7209) by filtering out sites based on the following criteria: a) a read mapping score higher than 40; b) minimum coverage greater than 10; and c) SNP genotypes called in > 90% of samples. Finally, a subset of high‐quality 5124500 SNPs was used for the genetic structure analysis. The population structure was analyzed by ADMIXTURE v1.3.0 with the pre‐defined genetic clusters increased from K = 1 to K = 5.^[^ ^75^ ^]^ Cross‐validation was used to identify the best‐fitting model of ADMIXTURE.

### Wing Loading

Body and thoracic mass were determined by weighing newly emergence adult moths on an electronic balance. The forewing of each moth was carefully removed at the wing base by using forceps and scissors. Using a digital caliper, the forewing length and forewing width were measured. The wing landmarks used for each measurement were as follows. Forewing length was from the base of the costal vein to the tip of the apical angle. Forewing width was from the apical end of the third radial branch to the analmargin (a line approximately parallel to the body). Wing‐area (cm^2^) was estimated using the equation Ln (Area) = (a+Ln (wing‐length*wing‐width)), where a is the intercept of the regression model, based on the Montgomery Equation.^[^ ^76^ ^]^ Wing‐loading (g cm^−2^) was estimated as body weight (g) / 2*wing area (cm^2^).^[^ ^77^ ^]^ For FAW‐mC strain, 82 adults were measured while 83 individuals were used in FAW‐mR strain.

### Flight Performance Assays

To exclude the possible interference of reproduction on flight performance only male adults were used for further studies.^[^ ^78^ ^]^ The FAW‐mC and FAW‐mR were separated and placed in glass culture dishes with moistened cotton. Following pupal emergence, male and female moths were prevented from mating, maintained separately within 30 cm × 30 cm × 30 cm cages, and fed on a daily basis with 10% honey water solution. Over three‐days old adults of FAW‐mC and FAW‐mR strain were subjected to flight performance assays. Flight performance (i.e., flight distance, duration, and speed) was assessed using a FXMD‐24‐USB flight mill (Jiaduo Science, Industry and Trade Co., Ltd., Henan, China). The flight mill was placed in a 70 cm × 50 cm × 114 cm MGC‐450HP artificial climate chamber (Yiheng Technology Instrument Co., Ltd., Shanghai, China) and FAW adults were individually attached to the apparatus. In detail, active and undamaged adults were carefully collected and stored individually within a 1.5‐mL cryopreservation tube. Next, the wings of diethyl ether‐anesthetized adults were expanded, scales were brushed from the intersect of abdomen and thorax, and tethering was done using a small droplet of 502 glue (Deli Group Co., Ltd., Zhejiang, China). After adult recovery, only undamaged and active individuals were attached to the flight mill. Next, the climate chamber was then completely darkened and moths were allowed to engage freely in tethered flight. Wingbeat frequency of tethered individuals was assessed three times before each flight test using a Phaser Strobe PBX stroboscope (Monado Monarch, Amherst, New Hampshire, USA). For each tested individual, Jiaduo Insect Fly Information System Software was used to convert specific recordings (e.g., number of mill revolutions per second) to different flight parameters such as total flight distance, duration and average speed. A total of 84 and 118 individual adults from FAW‐mC and FAW‐mR respectively were tested for flight performance.

### Cold Hardiness Assays

The supercooling point and freezing point of different development stages (3th instar larvae, 5th instar larvae and pupae) were determined using determination equipment (SUN‐V intelligent insect supercooling point tester; Pengcheng Electronic Technology Center of Beijing, China). The heat‐sensitive detector was inserted into a 1.5‐mL tube in direct contact with the cuticle of the insect and fixed in place with cotton around it. The tube was immersed in a refrigerated ethanol bath that was cooled at a rate of 1 °C min^−1^ (DW‐FL35 ultralow temperature freezing storage tank; Zhongke Meiling Low Temperature Technology Co., Ltd., Hefei, China). The temperature of the tested individual decreased with the environmental temperature. The supercooling point was reported as the lowest temperature before the insect body temperature increased as ice forms, and the freezing point was reported as the temperature at which ice crystals formed and endotherm and exotherm were in balance. For FAW‐mC strain, 38, 29, and 47 individuals of 3th, 5th, and pupa stage were measured while 38, 28, and 52 individuals were used in FAW‐mR strain.

### Fitness Comparison

To compare the lifespans of FAW‐mC and FAW‐mR strain individuals, the newly hatched neonates from egg masses were fed individually in plastic cups (4 cm in height by 5.6 cm in diameter) and provided daily with fresh artificial diet until pupation. One hundred newly hatched larvae were selected from FAW‐mC and FAW‐mR strains, respectively. The larval duration and the number of deaths were recorded until pupation. Each pupa was weighed on the first day after pupation and placed in a plastic cup (4 cm in height by 5.6 cm in diameter) until emergence and then the pupal duration was recorded. The pupae were mated (female: male = 1:1) in 500‐mL plastic cups bound with 120‐mesh gauze and rubber bands. All adults were fed daily with 10% (v/v) honey water. FAW adults that mated successfully included 44 and 37 pairs of the FAW‐mC and FAW‐mR strain, respectively. The pre‐oviposition period and the number of egg masses laid by each female daily were counted until the females died. All the FAW larvae and adults were placed in an artificial climate chamber with (25 ± 1) °C temperature, (60 ± 5) % RH and 16 h L:8 h D photoperiod. Moreover, the innate rate of increase (r) and finite rate of increase (λ) were further analyzed using the TWO‐SEX life table method.^[^ ^79^ ^]^

### Mitochondrial Genome Assembly

To compare the sequence difference of the mitochondrial genomes between FAW‐mC and FAW‐mR strains, 51 and 53 individuals were randomly selected to perform the whole genome sequencing using the Illumina NovaSeq 6000 platform with 150‐bp paired‐end reads. Then, the software NOVOPlasty v4.3.1 was used to extract and de novo assemble the mitochondrial DNA from the whole‐genome sequencing data.^[^ ^80^ ^]^ In detail, raw sequencing data were mapped to a mitogenome database using NextGenMap v0.5.5 with an identity threshold of 0.3. The sequences of FAW mitochondrial genome were downloaded from NCBI Sequence Database as candidate paired mitochondrial reads, in which at least one of the paired ones can be mapped, were extracted with SAMtools v1.7. Mitogenomes were assembled with a loop script that included the NOVOPlasty v4.3.1 assembler while storing has locally to speed up the assembly for possible multiple runs. Each mitogenome assembly required a seed or bait sequence, a short mitochondrial fragment used for the initial assembly. In practice, seeds can be generated using barcoding for each species or metabarcoding for multiplexed species pool (DNA libraries with a unique identifier for each species).

### Transmission Electron Microscopy

Flight muscle from FAW‐mC and FAW‐mR strains were dissected and fixed with 2.5% (wt/vol) glutaraldehyde for 1 h at room temperature and then treated with 1% OsO4 (Osmium tetroxide) in 0.1 m cacodylate buffer‐containing 0.1% CaCl2. After rinsing with cold distilled water, the tissues were dehydrated slowly with a grade series of ethanol concentration (from 30 to 100%) and embedded in Embed‐812 kit (EMS, PA). Ultrathin sections were performed with a diamond knife on an ULTRACUT UC7 ultramicrotome (Leica, Germany), deposited onto formvar‐coated slot grids, stained with 2% (wt/vol) phospyotungstic acid (PTA, pH 6.8). They were observed using a Tecnai G2 Spirit Twin transmission electron microscope (FEL, USA). Compacted and relaxed mitochondria taken from TEM images of flight muscle of different treatments were counted by using the analyses toolbox of Photoshop (Adobe Inc., San Jose, CA, USA). For each quantification, at least 4 moths were used for each treatment group. Statistical analyses were based on the average numbers for each FAW, and not based on total individual number of TEM images or organelle (mitochondria). When counting mitochondria present inside the flight muscle images, sampling was not performed. All mitochondria that are present in the images were calculated without bias or elimination. At least four randomly selected images of flight muscle were analyzed per FAW per treatment group and counting was performed for all mitochondria present in each image. Although the total number of mitochondria varies from image to image, the percentage of relaxed mitochondria was calculated per image and their averages determined the quantitative measure for each FAW.

### mtDNA Copy Number and ATP Assay

The copy number of mitochondria was detected using qPCR method. Flight muscle from FAW‐mC and FAW‐mR were dissected and DNA extracted using DNA Extraction Kits (Germany). One single‐copy Tpi gene (TpiF: AAATTTCTAGGTTGCCCATGCTC; TpiR: GCCTTAGTCTGCCTGAACAC) on the nuclear genome of FAW was used as a control, and the mtCO1 gene (COIF: GAGCTCCTGATATAGCTTTCCC; COIR: AGCTAAATCTACTGAACTACCACC) on the mitochondrial genome was used as mtDNA copy number marker genes. The 2^−△△ Ct^ method for relative quantification of gene expression was used to determine the relative mtDNA copy number. ATP content was measured using the ATP content assay kit (Solarbio BC0300), according to the manufacturer instructions. Briefly, flight muscle of five individuals from each strain (>3 days‐old adults) as one replicate were used to measure the ATP concentration. Eight repeats were performed for each strain. The luminescence was measured by Infinite F200 (Tecan, Swiss), and the results were compared to standards.

### In Silico Protein Effect Predictions

In order to explore the functional significance of the proposed sites that differed between the two strains, protein homology modeling was first used to visualize the potential functional implications. A sequence alignment was generated between human mitochondrial DNA (NC_012920) and the sequences that were generated for this study. Alignments were performed with CLC Genomics Workbench 22.0.2 (Qiagen, Inc.), and then mapped onto the human mitochondrial supercomplex PDB 5xth.^[^ ^81^ ^]^ Homology was lacking for the ND6 protein and therefore the study was unable to make predictions. Likewise, many amino acid substitutions were biochemically similar, and therefore any functional alterations that result would likely be subtle and difficult to conduct protein structure modeling. However, modeling of the amino acid changes in ND5 indicates they may result in changes in mitochondrial function. Molecular graphics and analyses were performed with UCSF Chimera, developed by the Resource for Biocomputing, Visualization, and Informatics at the University of California, San Francisco, with support from NIH P41‐GM103311.^[^ ^82^ ^]^ Amino acids were altered on the structure using the rotamer function in the structure editing command.

### Transcriptome Analysis

Total RNA was extracted from flight muscle (at least five replicates with five individuals per replicate) by using TRIzol reagent (Invitrogen). Complementary DNA libraries were generated using NEBNext UltraTM RNA Library Prep Kit for Illumina (NEB) following the manufacturer's recommendations, and index codes were added to attribute sequences to each sample. These analyses were conducted by Novogene (Tianjin, China). Raw data were filtered, and the cleaned data were mapped to the FAW genome sequence (http://v2.insect‐genome.com/Organism/715) with Bowtie2 v2.2.9 software^[^ ^83^ ^]^ and expression profiles determined as fragments per kilobase of transcript per million mapped reads (FPKM) by RSEM v1.3.0.^[^ ^84^ ^]^ Differentially expressed genes were analyzed by DESeq2 software.^[^ ^85^ ^]^ The threshold to determine the significant differentially expressed genes (DEGs) was ″fold change >1.5 and the p < 0.05. Gene Ontology (GO) and Kyoto Encyclopedia of Genes and Genomes (KEGG) enrichment analyses were conducted to determine the GO terms or KEGG pathways enriched in the DEGs. For GO functional enrichment, all the differentially expressed genes were mapped to the GO database (http://geneontology.org/) using Goatools (Version 0.6.5)^[^ ^86^ ^]^ with Fisher's exact test. KEGG enrichment analysis was carried out by R script based on the KEGG database (http://www.genome.jp/kegg/, Version 2017.08) with Fisher's exact test.^[^ ^87^ ^]^ RNA‐sequencing data were deposited in the Sequence Read Archive Database of NCBI (BioProject PRJNA1065590).

## Conflict of Interest

The authors declare no conflict of interest.

## Author Contributions

H.L., X.L., Y.P., Z.L., and L.Z. contributed equally to this work. The study was conceived and designed by Y.X., K.W., and H.L. Experiments were conducted by H.L., X.L., Y.P., P.W., and Z.L., and data analyses were mainly done by H.L. and Y.P. with contributions from some of the authors. The manuscript was written by H.L. and finalized by Y.X. with contributions from K.W., M.G., M.J., and L.Z. The submission has been approved by all relevant authors.

## Supporting information

Supporting Information

## Data Availability

The data that support the findings of this study are available in the supplementary material of this article.
