# Supplementary material for: Novel Mito‐Nuclear Combinations Facilitate the Global Invasion of a Major Agricultural Crop Pest
Source: Adv Sci (Weinh). 2024 Jul 4;11(34):2305353. doi: 10.1002/advs.202305353 (PMC11425838; doi:10.1002/advs.202305353)
Supplement: Supplementary file 1 — Supporting Information [file ADVS-11-2305353-s001.docx]

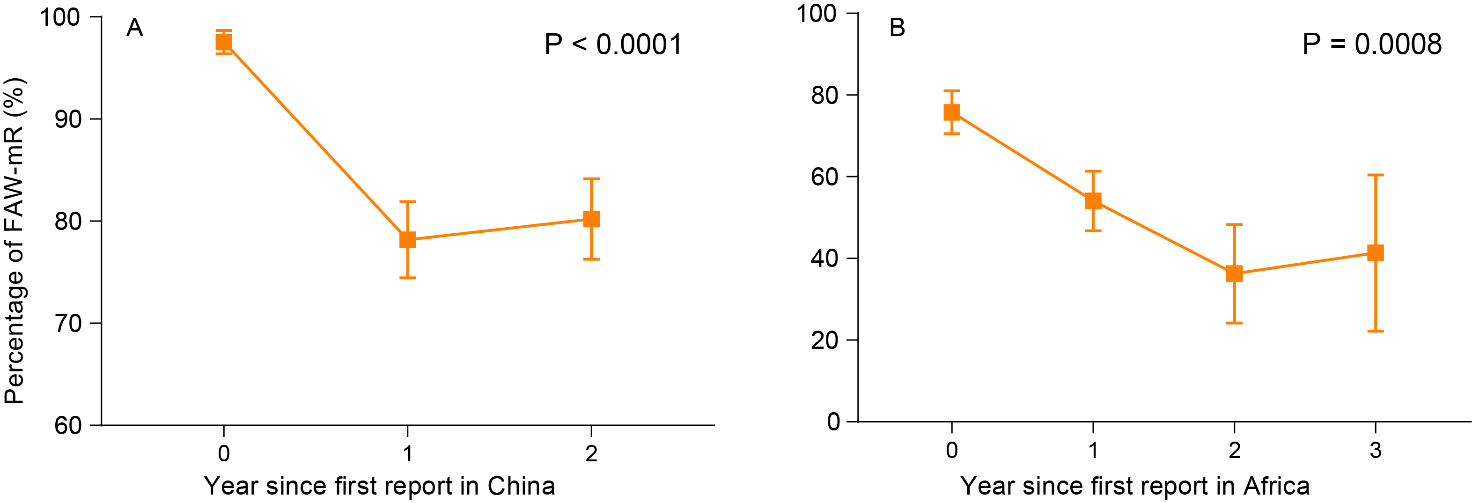


Figure S1. Effects of number of years since FAW were first reported in country at the point of collection in China and Africa. Data on *COI-RS* (FAW-mR) frequencies from Africa were extracted from Nagoshi et al. (2020) and Withers et al. (2021); data on year of first FAW report were extracted from data provided by the United Nations Food & Agriculture Organization website (https://www.fao.org/fall-armyworm/monitoring-tools/faw-map/en/).


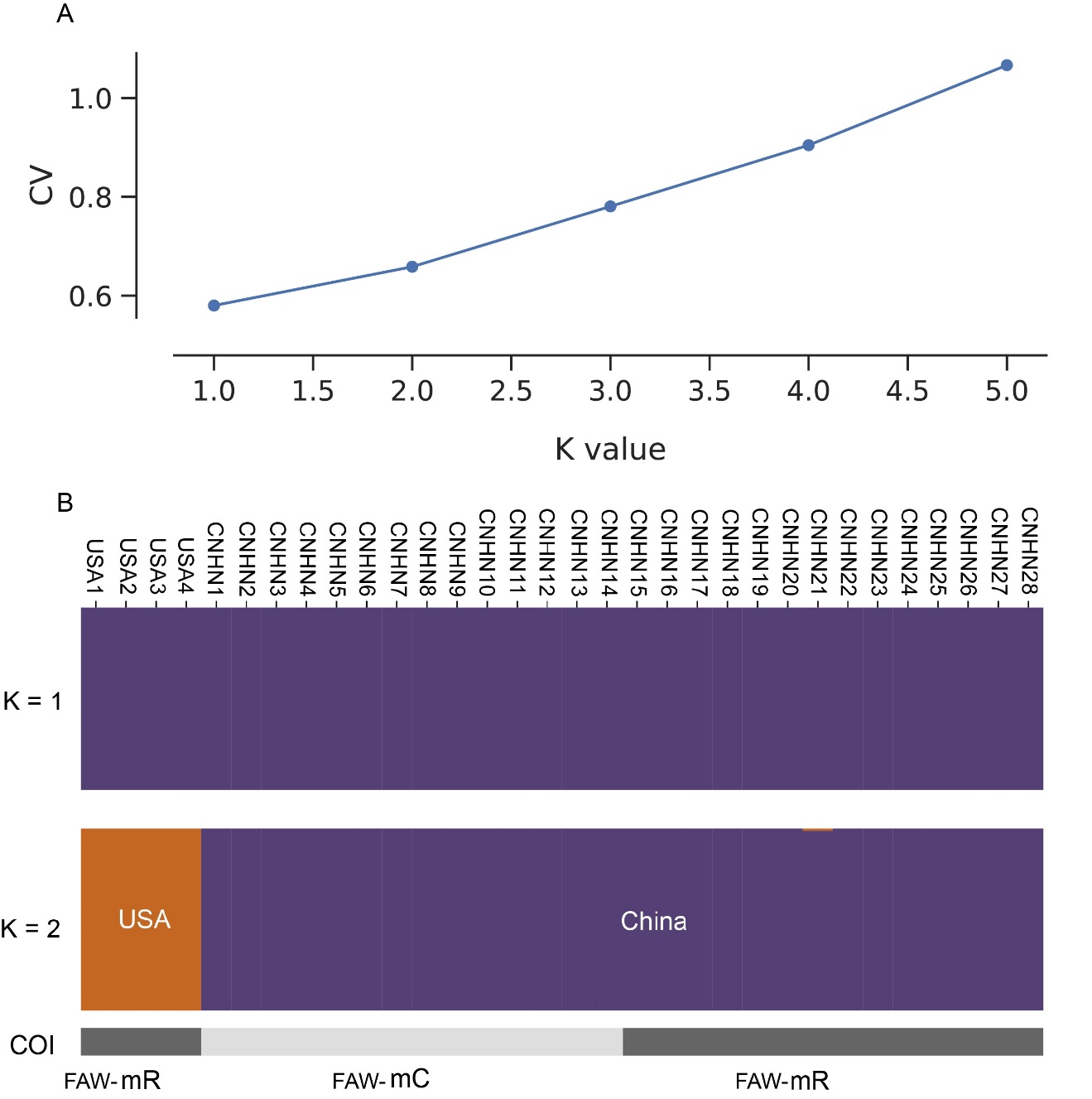


Figure S2. Genetic composition of FAW-mR and FAW-mC strains based on genome-wide SNPs. A, the estimated cross-validation error of possible clusters (K) from 1 to 5. B, genetic admixture of FAW-mR and FAW-mC strains. The color in each column indicates the proportion of individual in ancestral population. CNHN1- CNHN14 represents the FAW-mC individuals and CNHN1- CNHN14 represents the FAW-mR individuals in China. USA1-USA4 represents the FAW-mR individuals in USA.


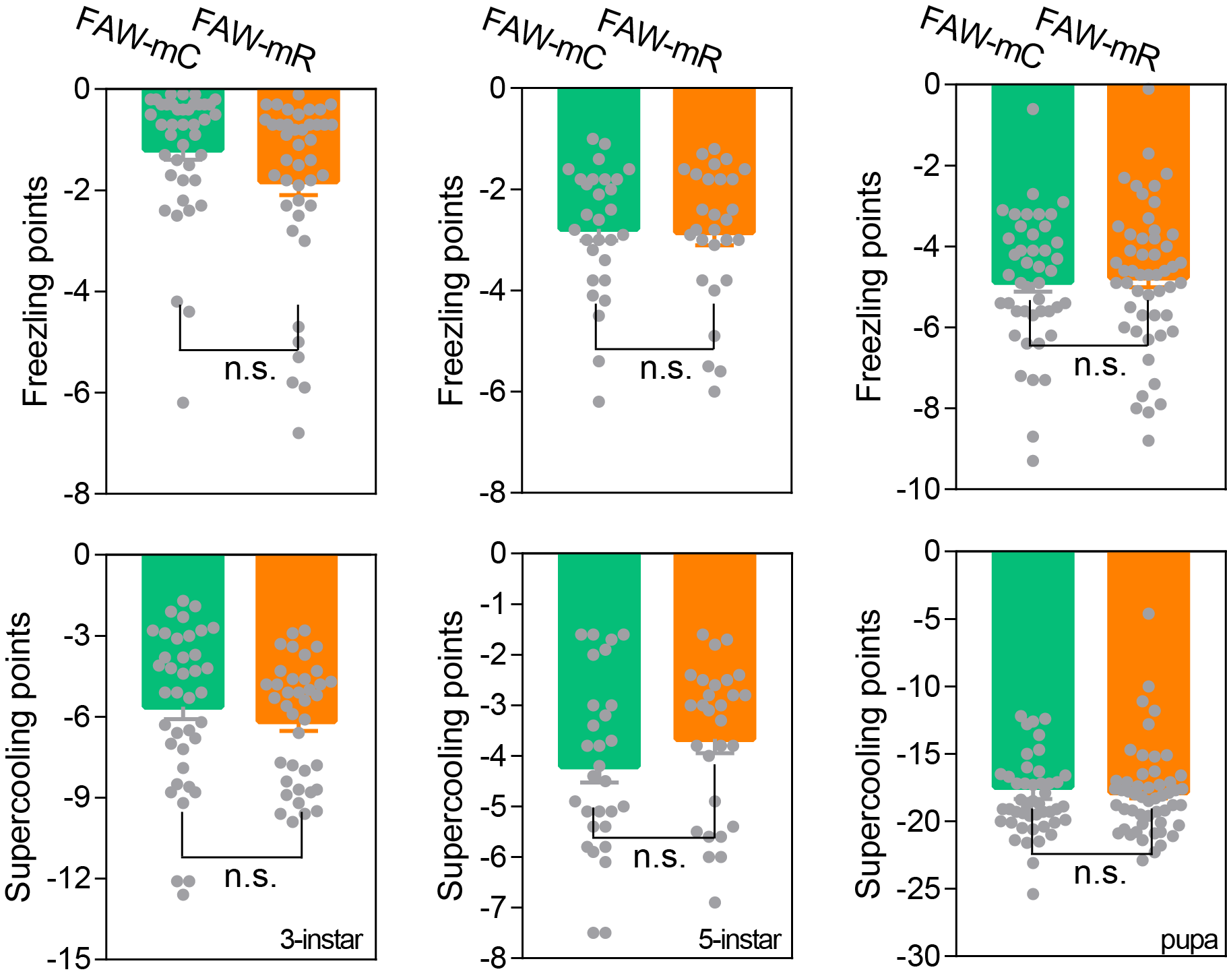


Figure S3. Cold hardiness of FAW-mC and FAW-mR strain in 3-instrar, 5-instar and pupa stage. “ns” indicate no significant differences.

Table S1. Detailed information of fall armyworm, *Spodoptera frugiperda* collecting from geographical locations across China in 2019

| **Province** | **Collection site** | **Longitude** | **Latitude** | **Collection date** | **FAW-mR** | **FAW-mR%** | **FAW-mC** | **FAW-mC%** |
| --- | --- | --- | --- | --- | --- | --- | --- | --- |
| Hubei | Xianning City,Hubei | 114.33 | 29.84 | 11/06/2019 | 2 | 100.00% | 0 | 0 |
|  | Xianning City,Hubei | 114.33 | 29.84 | 13/06/2019 | 2 |  | 0 |  |
|  | Yichang City, Hubei | 111.26 | 30.71 | 11/06/2019 | 2 |  | 0 |  |
|  | Yichang City, Hubei | 111.26 | 30.71 | 13/06/2019 | 2 |  | 0 |  |
|  | Xiaogan City, Hubei | 113.89 | 30.92 | 17/06/2019 | 2 |  | 0 |  |
| Guizhou | Guiyang City,Guizhou | 106.56 | 26.62 | 28/04/2019 | 5 | 100.00% | 0 | 0 |
|  | Guiyang City,Guizhou | 106.56 | 26.62 | 27/06/2019 | 2 |  | 0 |  |
| Chongqing | Chongqing | 106.69 | 29.46 | 07/06/2019 | 4 | 100.00% | 0 | 0 |
|  | Chongqing | 106.69 | 29.46 | 10/06/2019 | 2 |  | 0 |  |
| Zhejiang | Lishui City,Zhejiang | 119.95 | 28.44 | 18/05/2019 | 2 | 100.00% | 0 | 0 |
|  | Lishui City,Zhejiang | 119.95 | 28.44 | 25/05/2019 | 2 |  | 0 |  |
|  | Ningbo,Zhejiang | 121.49 | 29.86 | 01/06/2019 | 2 |  | 0 |  |
|  | Ningbo,Zhejiang | 121.49 | 29.86 | 03/06/2019 | 1 |  | 0 |  |
|  | Wenzhou City,Zhejiang | 120.63 | 27.99 | 01/06/2019 | 2 |  | 0 |  |
|  | Wenzhou City,Zhejiang | 120.63 | 27.99 | 03/06/2019 | 1 |  | 0 |  |
| Jiangxi | Jiujiang City,Jiangxi | 115.97 | 29.67 | 11/06/2019 | 7 | 100.00% | 0 | 0.00% |
|  | Jiujiang City,Jiangxi | 115.97 | 29.67 | 13/06/2019 | 2 |  | 0 |  |
|  | Ji'an City,Jiangxi | 114.94 | 27.12 | 07/06/2019 | 2 |  | 0 |  |
|  | Ji'an City,Jiangxi | 114.94 | 27.12 | 10/06/2019 | 2 |  | 0 |  |
|  | Nanchang City,Jiangxi | 115.78 | 28.67 | 11/06/2019 | 4 |  | 0 |  |
|  | Pingxiang City,Jiangxi | 113.82 | 27.64 | 07/06/2019 | 2 |  | 0 |  |
|  | Pingxiang City,Jiangxi | 113.82 | 27.64 | 10/06/2019 | 2 |  | 0 |  |
|  | Xinyu City,Jiangxi | 114.99 | 27.81 | 11/06/2019 | 2 |  | 0 |  |
| Guangdong | Guangzhou City,Guangdong | 113.47 | 23.15 | 23/04/2019 | 6 | 100.00% | 0 | 0.00% |
|  | Guangzhou City,Guangdong | 113.47 | 23.15 | 29/04/2019 | 3 |  | 0 |  |
|  | Huizhou City,Guangdong | 114.44 | 23.1 | 26/04/2019 | 2 |  | 0 |  |
|  | Jiangmen City,Guangdong | 113.12 | 22.51 | 29/04/2019 | 6 |  | 0 |  |
|  | Jiangmen City,Guangdong | 113.12 | 22.51 | 29/04/2019 | 1 |  | 0 |  |
|  | Zhanjiang City,Guangdong | 110.34 | 21.28 | 28/04/2019 | 5 |  | 0 |  |
|  | Zhanjiang City,Guangdong | 110.34 | 21.28 | 30/04/2019 | 2 |  | 0 |  |
| Guangxi | Baise City,Guangxi | 106.61 | 23.91 | 25/03/2019 | 2 | 93.75% | 0 | 6.25% |
|  | Baise City,Guangxi | 106.61 | 23.91 | 27/03/2019 | 1 |  | 0 |  |
|  | Hechi City,Guangxi | 108.01 | 24.71 | 11/03/2019 | 1 |  | 0 |  |
|  | Hechi City,Guangxi | 108.01 | 24.71 | 25/03/2019 | 0 |  | 1 |  |
|  | Hechi City,Guangxi | 108.01 | 24.71 | 01/04/2019 | 9 |  | 1 |  |
|  | Hechi City,Guangxi | 108.01 | 24.71 | 12/04/2019 | 4 |  | 0 |  |
|  | Qinzhou City,Guangxi | 108.59 | 21.95 | 10/04/2019 | 1 |  | 0 |  |
|  | Qinzhou City,Guangxi | 108.59 | 21.95 | 11/04/2019 | 6 |  | 0 |  |
|  | Yulin City,Guangxi | 110.11 | 22.67 | 10/04/2019 | 8 |  | 0 |  |
| Yunnan | Kunming City,Yunnan | 102.81 | 24.73 | 25/05/2019 | 1 | 100.00% | 0 | 0 |
|  | Kunming City,Yunnan | 102.81 | 24.73 | 27/05/2019 | 1 |  | 0 |  |
|  | Xishuangbanna Dai Autonomous Prefecture,Yunnan | 100.76 | 21.98 | 16/03/2019 | 5 |  | 0 |  |
|  | Ruili City,Yunnan | 97.78 | 23.97 | 30/07/2019 | 2 |  | 0 |  |
| Hainan | Haikou City,Hainan | 110.16 | 19.96 | 06/05/2019 | 2 | 100.00% | 0 | 0 |
|  | Haikou City,Hainan | 110.16 | 19.96 | 20/05/2019 | 2 |  | 0 |  |
|  | Sanya City,Hainan | 109.57 | 18.23 | 01/06/2019 | 2 |  | 0 |  |
|  | Lingao County,Hainan | 109.66 | 19.92 | 17/07/2019 | 1 |  | 0 |  |
| Gansu | Baiyin City,Gansu | 104.15 | 36.55 | 27/08/2019 | 2 | 100.00% | 0 | 0 |
|  | Longnan City,Gansu | 104.92 | 33.41 | 15/07/2019 | 4 |  | 0 |  |
| Henan | Luoyang City,Henan | 112.58 | 34.58 | 29/07/2019 | 1 | 100.00% | 0 | 0 |
|  | Nanyang City,Henan | 112.45 | 34.57 | 29/07/2019 | 1 |  | 0 |  |
|  | Yongcheng City,Henan | 116.69 | 33.94 | 05/08/2019 | 2 |  | 0 |  |
| Shandong | Liaocheng City,Shandong | 115.86 | 36.45 | 27/08/2019 | 2 | 100.00% | 0 | 0 |
|  | Tengzhou City,Shandong | 117.08 | 35.09 | 30/07/2019 | 2 |  | 0 |  |
|  | Changdao County, Shandong | 120.55 | 38.24 | 06/09/2019-03/11/2019 | 65 |  | 0 |  |
| Anhui | Xuancheng City,Anhui | 118.77 | 30.96 | 01/07/2019 | 2 | 100.00% | 0 | 0 |
|  | Xuancheng City,Anhui | 118.77 | 30.96 | 01/07/2019 | 2 |  | 0 |  |

Table S2. Detailed information of fall armyworm, *Spodoptera frugiperda* collecting from geographical locations across China in 2020

| **Province** | **Collection site** | **Longitude** | **Latitude** | **Collection date** | **FAW-mR** | **FAW-mR%** | **FAW-mC** | **FAW-mC%** |
| --- | --- | --- | --- | --- | --- | --- | --- | --- |
| Anhui | Anqing City, Anhui | 117.05 | 30.53 | 20/06/2020 | 2 | 88.37% | 0 | 11.63% |
|  | Suzhou City,Anhui | 116.96 | 33.65 | 27/08/2020 | 4 |  | 1 |  |
|  | Tongling City,Anhui | 117.84 | 30.92 | 28/08/2020 | 29 |  | 3 |  |
|  | Wuhu City,Anhui | 118.41 | 31.35 | 15/09/2020 | 3 |  | 1 |  |
| Sichuan | Luzhou City,Sichuan | 105.46 | 28.85 | 15/05/2020 | 0 | 45.45% | 2 | 54.55% |
|  | Liangshan Yi Autonomous Prefecture,Sichuan | 102.24 | 27.87 | 22/5/2020-2/6/2020 | 10 |  | 10 |  |
| Guizhou | Southwest Guizhou Autonomous Prefecture | 104.91 | 25.08 | 03/03/2020 | 5 | 58.70% | 15 | 41.30% |
|  | Guiyang City,Guizhou | 106.63 | 26.64 | 09/06/2020 | 15 |  | 3 |  |
|  | Anshun City,Guizhou | 105.94 | 26.26 | 14/05/2020 | 3 |  | 0 |  |
|  | Zunyi City,Guizhou | 106.92 | 27.70 | 14/05/2020 | 3 |  | 0 |  |
|  | Guiyang City,Guizhou | 106.63 | 26.64 | 05/07/2020 | 1 |  | 1 |  |
| Guangdong | Shaoguan City,Guangdong | 113.62 | 24.80 | 13/05/2020 | 4 | 71.79% | 0 | 28.21% |
|  | Jieyang City,Guangdong | 116.37 | 23.57 | 20/05/2020 | 2 |  | 1 |  |
|  | Huizhou City,Guangdong | 114.37 | 23.11 | 20/05/2020 | 3 |  | 1 |  |
|  | Huizhou City,Guangdong | 114.42 | 23.10 | 14/07/2020 | 2 |  | 1 |  |
|  | Yangjiang City,Guangdong | 111.99 | 21.85 | 10/08/2020 | 8 |  | 6 |  |
|  | Jiangmen City,Guangdong | 113.08 | 22.58 | 19/08/2020 | 9 |  | 2 |  |
| Gunagxi | Yulin City,Guangxi | 110.17 | 22.67 | 21/05/2020 | 1 | 77.94% | 1 | 22.06% |
|  | GuigangCity,Guangxi | 109.61 | 23.11 | 22/05/2020 | 3 |  | 1 |  |
|  | Beihai City,Guangxi | 109.12 | 21.47 | 09/07/2020 | 2 |  | 0 |  |
|  | Beihai City,Guangxi | 109.09 | 21.47 | 20/07/2020 | 0 |  | 2 |  |
|  | Qinzhou City,Guangxi | 108.65 | 21.98 | 20/07/2020 | 1 |  | 1 |  |
|  | Laibing City,Guangxi | 109.23 | 23.76 | 27/07/2020 | 2 |  | 0 |  |
|  | Guilin City,Guangxi | 110.31 | 25.27 | 14/09/2020 | 5 |  | 1 |  |
|  | Nanning City,Guangxi | 108.38 | 22.85 | 31/12/2020 | 39 |  | 9 |  |
| Yunnan | Yunshan City,Yunnan | 99.95 | 22.55 | 20/06/2020 | 2 | 79.20% | 0 | 20.80% |
|  | Pu'er City,Yunnan | 100.96 | 22.82 | 2/1/2020-4/6/2020 | 97 |  | 26 |  |
|  | Yunshan City,Yunnan | 102.70 | 25.04 | 05/08/2020 | 14 |  | 5 |  |
|  | Pu'er City,Yunnan | 100.97 | 22.82 | 07/12/2020 | 75 |  | 36 |  |
| Hebei | Shijiazhuang City,Hebei | 114.35 | 38.03 | 23/08/2020 | 6 | 100.00% | 0 | 0 |
|  | Xintai City,Hebei | 114.56 | 37.09 | 01/09/2020 | 8 |  | 0 |  |
|  | Handan City,Hebei | 114.55 | 36.63 | 31/08/2020 | 3 |  | 0 |  |
| Gansu | Baiyin City,Gansu | 104.11 | 36.54 | 28/08/2020 | 7 | 100.00% | 0 | 0 |
| Hubei | Shiyan City,Hubei | 110.80 | 32.62 | 08/07/2020 | 1 | 65.00% | 1 | 35.00% |
|  | Yichang City,Hubei | 111.30 | 30.70 | 07/07/2020 | 2 |  | 0 |  |
|  | Wuxue City,Hubei | 115.49 | 29.89 | 07/08/2020 | 0 |  | 2 |  |
|  | Wuhan City,Hubei | 114.32 | 30.56 | 27/08/2020 | 1 |  | 2 |  |
|  | Jingmen City,Hubei | 112.18 | 31.03 | 16/09/2020 | 2 |  | 0 |  |
|  | Shiyan City,Hubei | 110.82 | 32.62 | 27/09/2020 | 7 |  | 2 |  |
| Chongqing | Chongqing | 106.60 | 29.55 | 23/07/2020 | 10 | 85.00% | 0 | 15.00% |
|  | Chongqing | 106.51 | 29.62 | 29/07/2020 | 2 |  | 0 |  |
|  | Chongqing | 106.59 | 29.54 | 20/08/2020 | 5 |  | 3 |  |
| Zhejiang | Wenzhou City, Zhejiang | 120.70 | 28.00 | 22/07/2020 | 14 | 90.00% | 2 | 10.00% |
|  | Ningbo City,Zhejiang | 121.56 | 29.89 | 25/08/2020 | 4 |  | 0 |  |
| Jinagxi | Xinyu City,Jiangxi | 114.89 | 27.80 | 31/08/2020 | 12 | 92.31% | 1 | 7.69% |
| Fujian | Zhangzhou City,Fujian | 117.64 | 24.52 | 21/07/2020 | 4 | 82.81% | 0 | 17.19% |
|  | Zhangzhou City,Fujian | 117.64 | 24.52 | 07/12/2020 | 36 |  | 10 |  |
|  | Yongan City,Fujian | 117.35 | 25.93 | 22/07/2020 | 4 |  | 1 |  |
|  | Putian City,Fujian | 118.99 | 25.45 | 22/07/2020 | 9 |  | 0 |  |
| Hunan | Yongzhou City,Hunan | 111.63 | 26.41 | 23/07/2020 | 2 | 85.00% | 0 | 15.00% |
|  | Zhuzhou City,Hunan | 113.16 | 27.83 | 23/07/2020 | 2 |  | 0 |  |
|  | Jianghua City,Hunan | 111.57 | 25.18 | 23/07/2020 | 13 |  | 3 |  |

| **Province** | **Collection site** | **Longitude** | **Latitude** | **Collection date** | **FAW-mR** | **FAW-mR%** | **FAW-mC** | **FAW-mC%** |
| --- | --- | --- | --- | --- | --- | --- | --- | --- |
|  |  |  |  |  |  |  |  |  |
| Yunnan | Pu'er City,Yunnan | 101.86 | 22.58 | 2021/4/4 | 20 | 74.07% | 7 | 25.93% |
|  | Pu'er City,Yunnan | 100.97 | 22.82 | 2021/6/25 | 17 | 44.74% | 21 | 55.26% |
|  | Pu'er City,Yunnan | 101.87 | 22.58 | 03/10/2021 | 10 | 47.62% | 11 | 52.38% |
|  |  |  |  |  |  |  |  |  |
| Hainan | Sanya City,Hainan | 109.52 | 18.24 | 2021/1/9 | 70 | 58.33% | 50 | 41.67% |
|  | Hainan | 109.56 | 19.54 | 2021/3/12 | 14 | 70.00% | 6 | 30.00% |
|  | Lingshui City,Hainan | 110.03 | 18.48 | 22/09/2021 | 16 | 51.61% | 15 | 48.39% |
|  |  |  |  |  |  |  |  |  |
| Shanxi | Yuncheng City,Shanxi | 111.03 | 35.05 | 31/07/2021 | 11 | 100.00% | 0 | 0.00% |
|  |  |  |  |  |  |  |  |  |
| Shandong | Changdao County, Shandong | 120.55 | 38.24 | 21/8/2021 | 4 | 100.00% | 0 | 0.00% |
|  |  |  |  |  |  |  |  |  |
| Shannxi | Hanzhong city, Shannxi | - | - | 16/07/2021 | 27 | 100.00% | 0 | 0.00% |
|  |  |  |  |  |  |  |  |  |
| Hubei | Xianning City,Hubei | 114.32 | 29.84 | 2021/6/1 | 34 | 100.00% | 0 | 0.00% |
|  | Wuxue City,Hubei | 115.54 | 29.87 | 01/07/2021 | 46 | 97.87% | 1 | 2.13% |
|  | Jingzhou City,Hubei | - | - | 01/07/2021 | 21 | 100.00% | 0 | 0.00% |

Table S3. Detailed information of fall armyworm, *Spodoptera frugiperda* collecting from geographical locations across China in 2021

Table S4. Quantification of relaxed mitochondria percentage

| Treatment group | Strain | |
| --- | --- | --- |
|  | FAW-mC | FAW-mR |
| Number of FAW | 4 | 4 |
| Total number of muscle images counted | 19 | 19 |
| Total number of mitochondria counted in all images | 1167 | 1354 |
